# Supplementary material for: COVID-19 Biomarkers Comparison: Children, Adults and Elders
Source: Medicina (Kaunas). 2023 May 3;59(5):877. doi: 10.3390/medicina59050877 (PMC10222231; doi:10.3390/medicina59050877)
Supplement: Supplementary file 1 [file medicina-59-00877-s001.zip › medicina-2365707-supplementary.pdf]

Supplementary material no. 1

Visually, the ROC curves for each biomarker, in regards to ICU admission, can be observed in figures 1-6. Figure 7-12 show the ROC curves for each biomarker, in regards to the deceased.

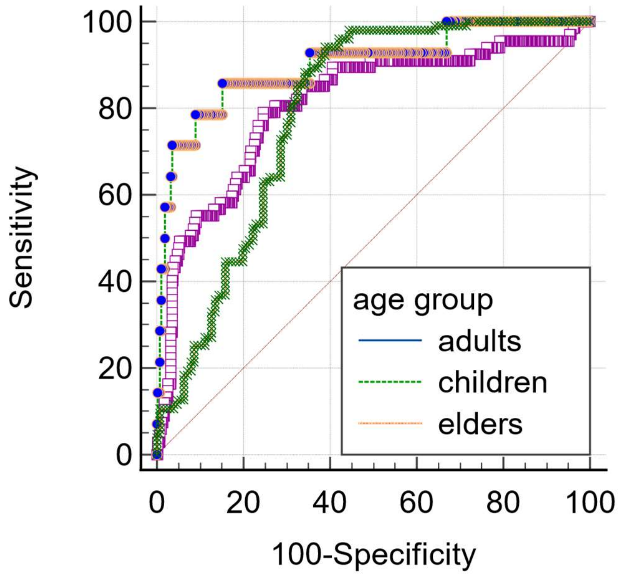

Figure 1. ROC curve analysis and comparison of CRP in regards to ICU admission

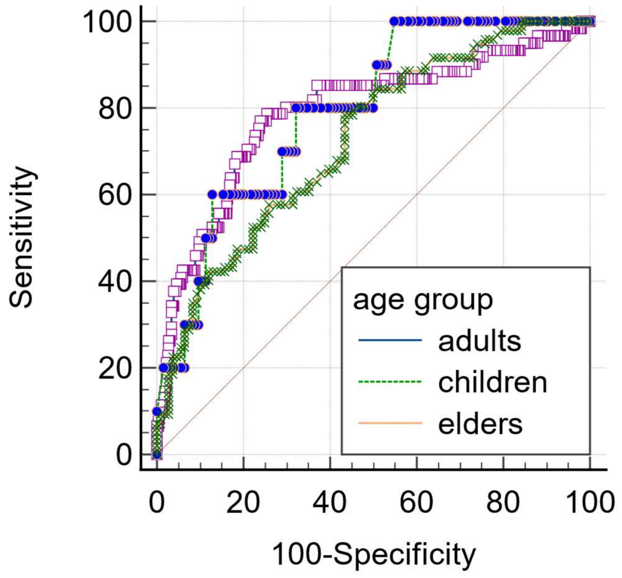

Figure 2. ROC curve analysis and comparison of LDH in regards to ICU admission

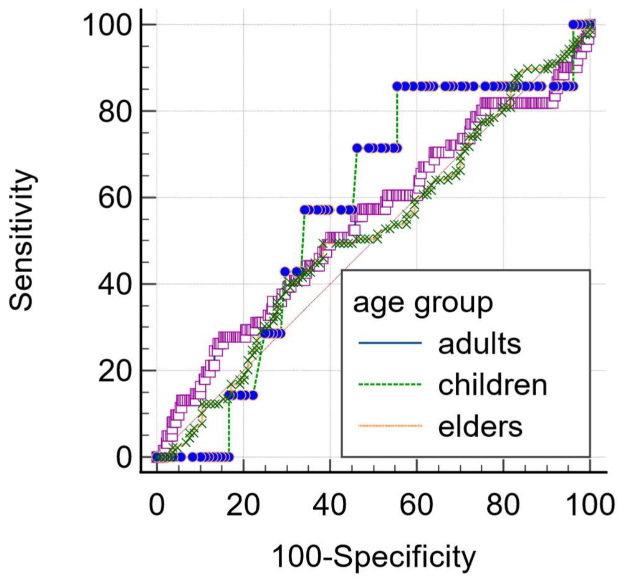

Figure 3. ROC curve analysis and comparison of CK in regards to ICU admission

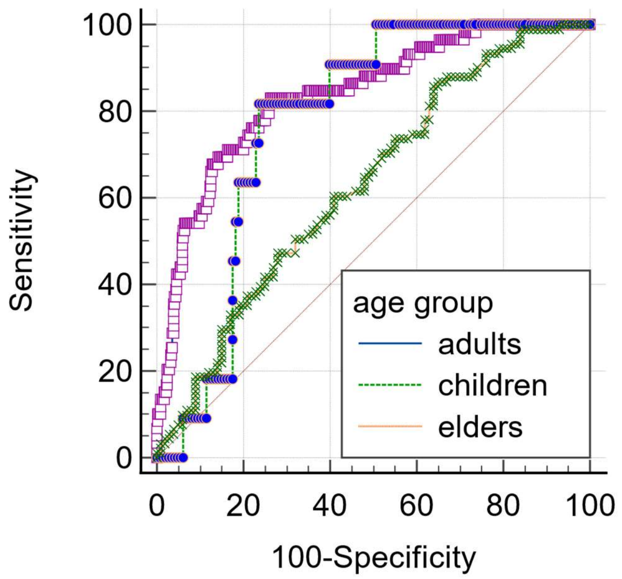

Figure 4. ROC curve analysis and comparison of vitamin D in regards to ICU admission

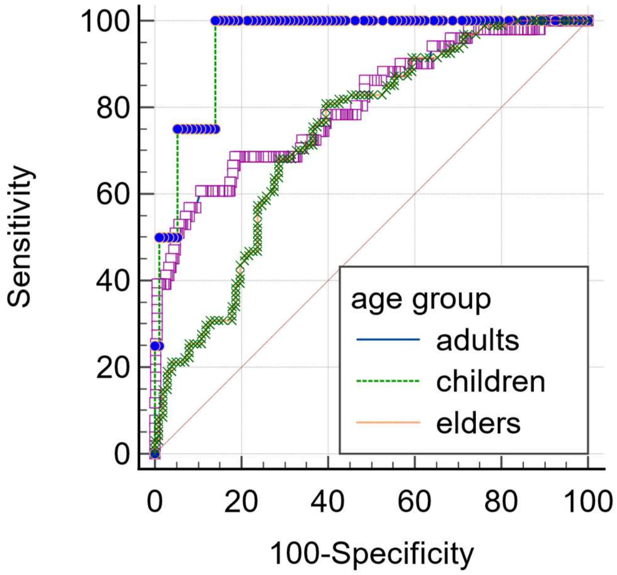

Figure 5. ROC curve analysis and comparison of ferritin in regards to ICU admission

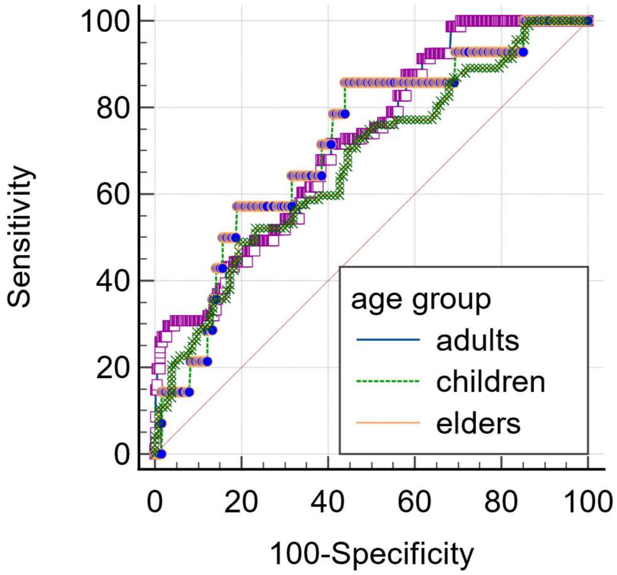

Figure 6. ROC curve analysis and comparison of HDL in regards to ICU admission

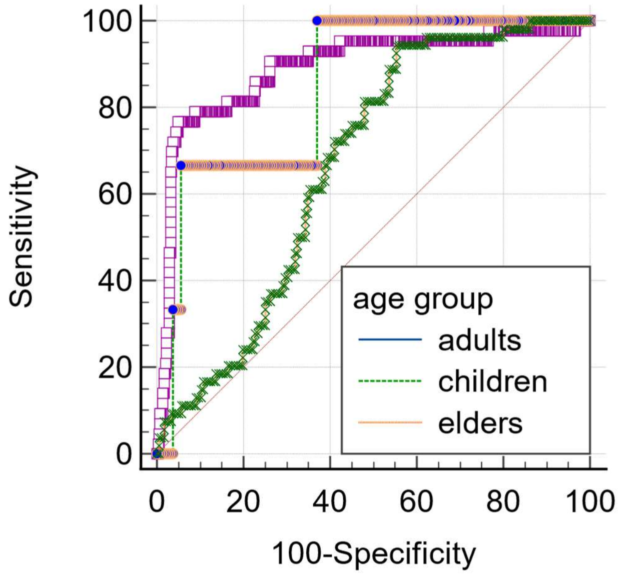

Figure 7. ROC curve analysis and comparison of CRP in regards to death

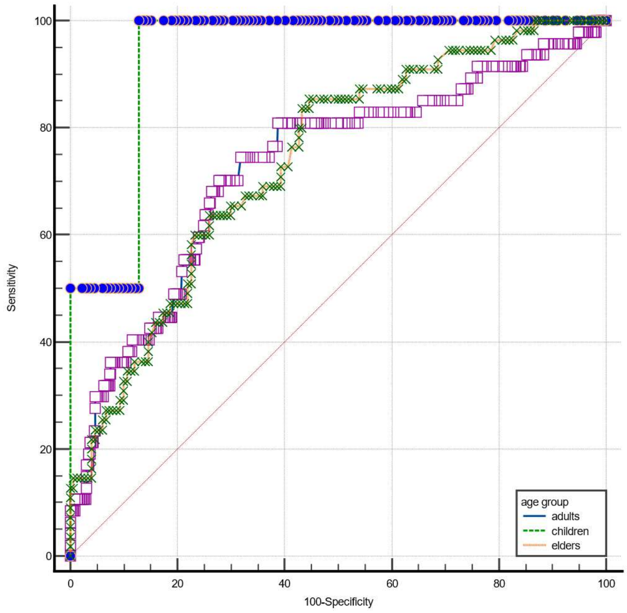

Figure 8. ROC curve analysis and comparison of LDH in regards to death

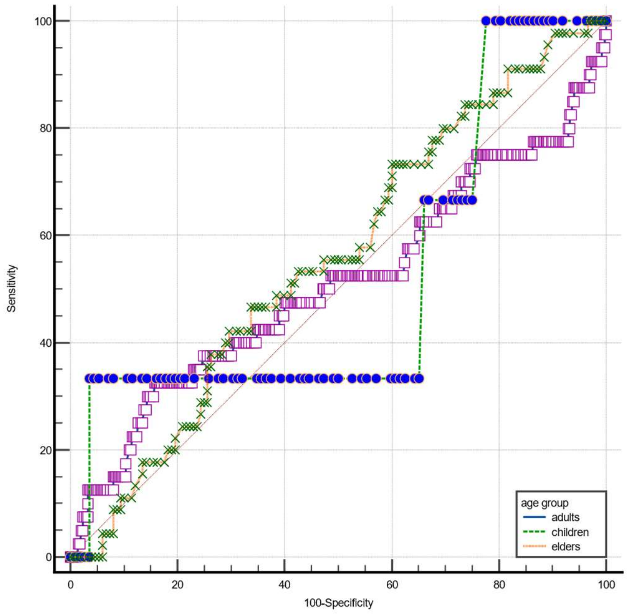

Figure 9. ROC curve analysis and comparison of CK in regards to death

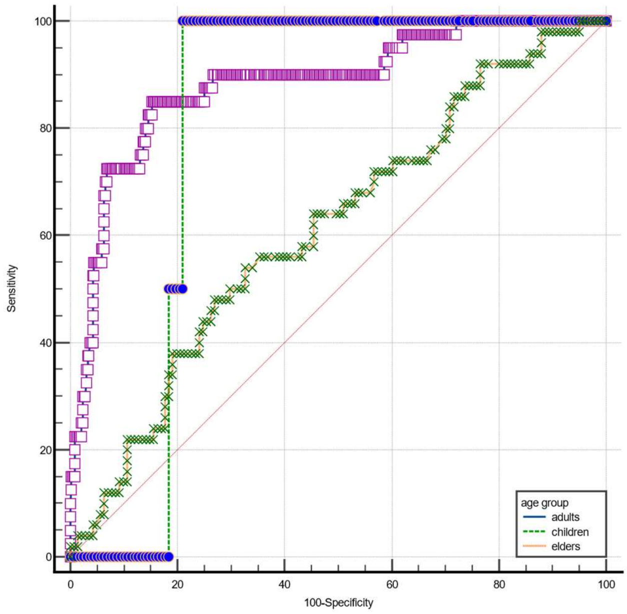

Figure 10. ROC curve analysis and comparison of vitamin D in regards to death

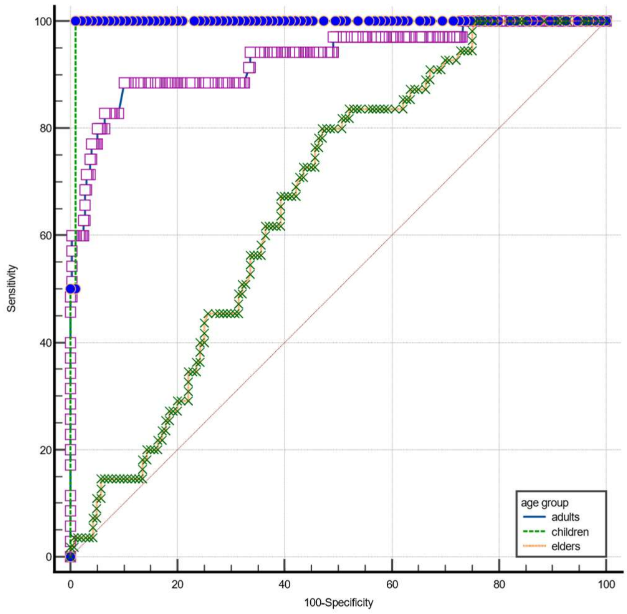

Figure 11. ROC curve analysis and comparison of ferritin in regards to death

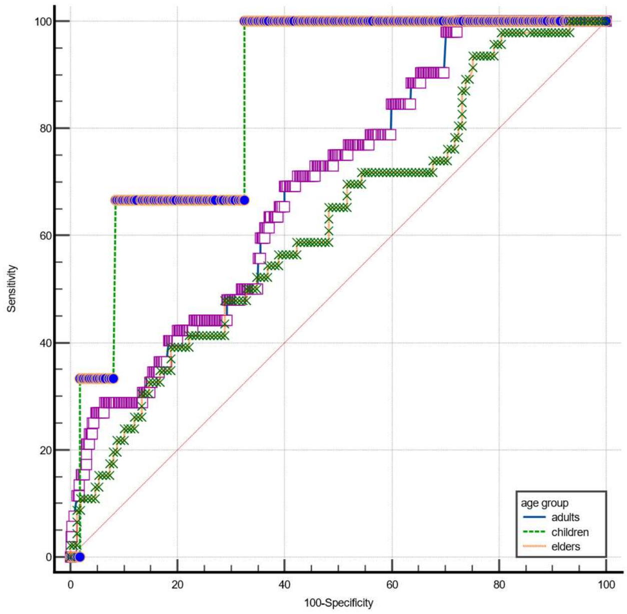

Figure 12. ROC curve analysis and comparison of HDL in regards to death
